# Subnational mapping for targeting anaemia prevention in women of reproductive age in Ethiopia: A coverage‐equity paradox

**DOI:** 10.1111/mcn.13277

**Published:** 2021-10-08

**Authors:** Bayuh Asmamaw Hailu, Arnaud Laillou, Stanley Chitekwe, Joseph Beyene, Kaleab Baye

**Affiliations:** ^1^ Monitoring and Evaluation Wollo University Dessie Ethiopia; ^2^ Nutrition Section UNICEF Ethiopia Addis Ababa Ethiopia; ^3^ Department of Health Research Methods, Evidence, and Impact McMaster University Canada; ^4^ Center for Food Science and Nutrition, College of Natural and Computational Sciences Addis Ababa University Addis Ababa Ethiopia; ^5^ Research Center for Inclusive Development in Africa (RIDA) Addis Ababa Ethiopia

**Keywords:** anaemia, effective coverage, inequality, severe anaemia, women

## Abstract

Anaemia in women of reproductive age (WRA) can be effectively addressed if supported by a better understanding of the spatial variations, magnitude, severity and distribution of anaemia. This study aimed to map the subnational spatial distribution of anaemia (any, moderate and severe forms) among WRA in Ethiopia. We identified and mapped (any, moderate and severe) anaemia hotspots in WRA (*n* = 14,923) at the subnational level and identified risk factors using multilevel logistic regression. Kulldorff scan statistics were used to identify hotspot regions. Ordinary kringing was used to predict the anaemia prevalence in unmeasured areas. The overall anaemia prevalence increased from 16.6% in 2011 to 23.6% in 2016, a rise that was mostly related to the widening of existing hotspot areas. The primary clusters of (any) anaemia were in Somali and Afar regions. The horn of the Somali region represented a cluster of 330 km where 10% of WRA were severely anaemic. The Oromia–Somali border represented a significant cluster covering 247 km, with 9% severe anaemia. Population‐dense areas with low anaemia prevalence had high absolute number of cases. Women education, taking iron‐folic‐acid tablets during pregnancy and birth‐delivery in health facilities reduced the risk of any anaemia (*P* < 0.05). The local‐level mapping of anaemia helped identify clusters that require attention but also highlighted the urgent need to study the aetiology of anaemia to improve the effectiveness and safety of interventions. Both relative and absolute anaemia estimates are critical to determine where additional attention is needed.

**Key messages**

- Contrary to the World Health Assembly's target, anaemia increased in Ethiopian women.
- Both relative and absolute anaemia estimates are critical to prioritize interventions.
- Anaemia distribution shows clustering in a handful of clusters.

## 1 INTRODUCTION

Anaemia is highly prevalent in low‐and‐middle income countries (LMICs) and disproportionately affects women of reproductive age (WRA) (15–45 years of age) and young children (Chaparro & Suchdev, 2019). Anaemia adversely affects the health and well‐being of WRA, and it compromises cognitive function and reduces productivity (Cook et al., 2017; Scholz et al., 1997). Besides, anaemia is associated with adverse perinatal outcomes and maternal mortality. Consequently, reducing anaemia in WRA by 50% by 2025 was set as one of the six World Health Assembly (WHA) targets and was then endorsed as one of the Sustainable Development Goals (SDGs) target for 2030.

The aetiology of anaemia is complex and includes micronutrient deficiencies, malaria, inadequate water, sanitation and hygiene (WASH), hemoglobinopathies, and chronic diseases (Chaparro & Suchdev, 2019). However, many earlier studies have estimated that about 50% of anaemia in WRA is associated with iron deficiency, and this has led to iron interventions becoming the main and often the only intervention delivered for the prevention and treatment of anaemia (Stevens et al., 2013). Recent studies have contested this assumption and highlighted that the magnitude and cause of anaemia is context dependent, but World Health Organization (WHO) advocates for iron‐folic acid provision for WRA living in settings where the prevalence of anaemia is 20% or higher (Petry et al., 2016; WHO, 2014; Wieringa et al., 2016). This cut‐off of 20% is often used to make decisions at the national level, hence undermining local variations in the magnitude and causes of anaemia.

A subnational estimate of anaemia in WRA is critical to monitor progress, but also target interventions to reach the most vulnerable segments of the population. Anaemia prevention and treatment could be more effective if the spatial variations, magnitude, severity and distribution were characterized. Such local‐level mapping has been generated for WHA targets like stunting, wasting and overweight, but not for anaemia (Kinyoki et al., 2020; Osgood‐Zimmerman et al., 2018). Ethiopia has endorsed the WHA and SDG targets of reducing anaemia in WRA by 50% relative to 2012 baseline figures (FDRE, 2016). However, according to the WHO global targets tracking tool (https://www.who.int/tools/global-targets-tracking-tool), and the recent WHO estimates (WHO, 2021), Ethiopia is off‐track from meeting the anaemia targets. Consequently, effective interventions that prevent and treat anaemia among WRA are urgently needed.

Therefore, the present study aimed to map the spatial distribution of anaemia (any, moderate and severe forms) among WRA in Ethiopia to support the design and implementation of effective anaemia prevention interventions. We, therefore, identified and mapped anaemia hotspots at the subnational level and identified risk factors. Given that Ethiopia has not started a widespread food fortification with nutrients, our results are not confounded by the access and consumption of fortified foods. This study can inform the design and prioritization of interventions aiming to prevent/treat anaemia among WRA and can show within‐country inequalities, and contribute towards achieving nutrition goals of the WHA and the SDGs.

## 2 METHODS

### 2.1 Overview and data source

We estimated and mapped the prevalence of any, moderate and severe forms of anaemia in WRA (15–49 years of age), using the latest two rounds of the Ethiopian Demographic and Health Surveys (EDHS 2011–2016) (CSA & ICF, 2011, 2016). Enumeration areas where the haemoglobin concentrations were taken were linked to the geographical coordinates using global positioning system (GPS). The subnational prevalence of any, moderate and severe anaemia was estimated and mapped. Spatial heterogeneity analysis was conducted using Kulldorff scan statistics and hotspot regions were identified (Kulldorff, 1997). The prevalence of any, moderate and severe anaemia was predicted using ordinary kringing for unmeasured areas. Finally, using multilevel logistic regression, factors associated with any and severe forms of anaemia were identified. A mixed‐effect logistic regression model was run, and adjusted odds ratios (AORs) with corresponding 95% confidence intervals (CI) were estimated.

### 2.2 Outcomes

The outcomes of interest were any, moderate and severe anaemia in WRA (15–49 years of age), which were defined as altitude‐adjusted haemoglobin concentrations of <12.0 g/dl (<11.0 g/dl for pregnant women), 8.0–10.9 g/dl (7.0–10.9 g/dl for pregnant) and <8.0 g/dl (7.0 g/dl for pregnant), respectively (WHO, 2011).

### 2.3 Statistical analysis

#### 2.3.1 Spatial statistics

Hotspots and coldspots were identified using local G* statistics. Based on 95% CI, hotspots were defined as areas with anaemia prevalence with a z‐score ≥ 2 and cold‐spots as areas with a z‐score ≤ −2 (*P* < 0.05).

Kulldoruff's scan statistic was used to quantify the spatial distribution of the prevalence of any, moderate, and severe anaemia (Kulldorff, 1997). A purely spatial scan statistic was used to identify areas with higher anaemia cases than would have been predicted if the risk of anaemia was uniformly distributed (Jung et al., 2007). Spatially important higher and lower aggregate concentrations were identified and were represented by circular windows. Finally, confirmatory spatial analysis was run using SATScan and QGIS by applying purely spatial Poisson scan statistics. With the discrete Poisson model, the number of cases in each cluster (enumeration area) was estimated (Jung et al., 2007; Kulldorff & Nagarwalla, 1995; Kutoyants, 2012).

To forecast the prevalence of anaemia from unmeasured areas, spatial interpolation using ordinary kriging was applied using SAGA GIS (Stein, 2012).

#### 2.3.2 Multilevel logistic regression

Multilevel logistic regression was run to assess factors associated with clustering of anaemia prevalence. Variables with *P* < 0.2 in the univariate analysis were included in the multiple regression. Considering the hierarchical nature of the DHS data, we run a multilevel logistic regression at the individual, household and community levels (Hox et al., 2017). Four models containing social and biologically relevant variables have been constructed. The first model (M0) is a null model without independent variables to measure random variability using the Intra Community Correlation (ICC). The ICC was evaluated to determine whether the difference in cases is mainly within or between households (Diez, 2002). The second model (M1) was adapted to all lower level (individual level) factors; the third model (M2) was used for all higher level factors; and the fourth model (M3) accounted both lower and higher‐level factors. Model goodness‐of‐fit was checked by the Akaike Information Criterion (AIC). Statistical analyses were conducted using Stata v14.

## 3 RESULTS

**Figure 1:**
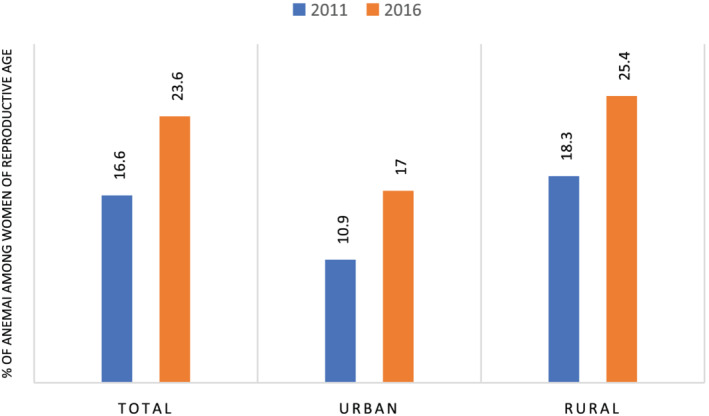
Prevalence of anaemia among women of reproductive age, 2011 and 2016

**Figure 2:**
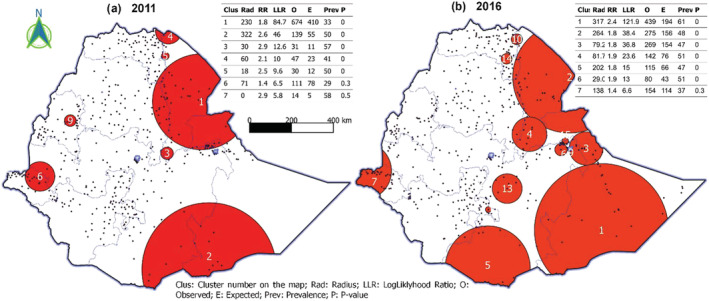
Clusters of any anaemia among women of reproductive age, 2011 and 2016

**Figure 3:**
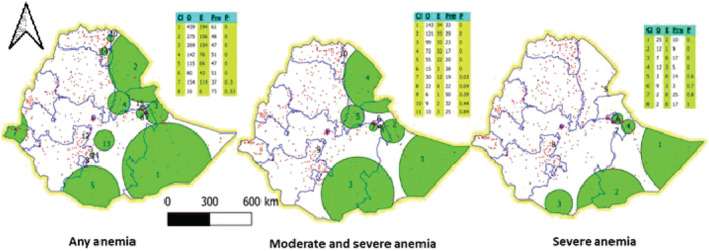
Clusters of anaemia among women of reproductive age by severity, 2016

Our analyses included a total of 14,923 WRA (15–49 years of age). The anaemia prevalence increased from 16.6% in 2011 to 23.6% in 2016 (Figure 1). The increase (~7%) was proportional for both urban and rural areas. The clusters with high prevalence of any anaemia had increased in number between 2011 and 2016 (Figure 2). Further disaggregating anaemia cases by severity, Figure 3 shows significant clusters of (any) anaemia spread across the eastern part of Ethiopia, with few smaller clusters in the central, southern, and western part of the country. In contrast, clusters of severe anaemia were almost entirely localized in the southern and eastern part of Ethiopia.

The primary clusters of (any) anaemia were found in Somali and Afar region. A cluster of 318 km radius was found in the Somali region, where 33% of WRA were anaemic with a relative risk (RR) of 2.4 (*P* < 0.001; Tables S1 & S2). The second important cluster was 265 km wide and found in the Afar region, where 50% of WRA were anaemic with a RR of 1.8 (*P* < 0.001). The Somali region was also a primary cluster for severe forms of anaemia. The horn of the Somali region represented a cluster of severe anaemia that spanned 330 km wide and where 10% (RR: 3.5; *P* < 0.001) of WRA were severely anaemic. The Oromia–Somali region's border also represented a significant cluster covering 247 km, with 9% of WRA with severe anaemia (RR: 5; *P* < 0.001).

**Figure 4:**
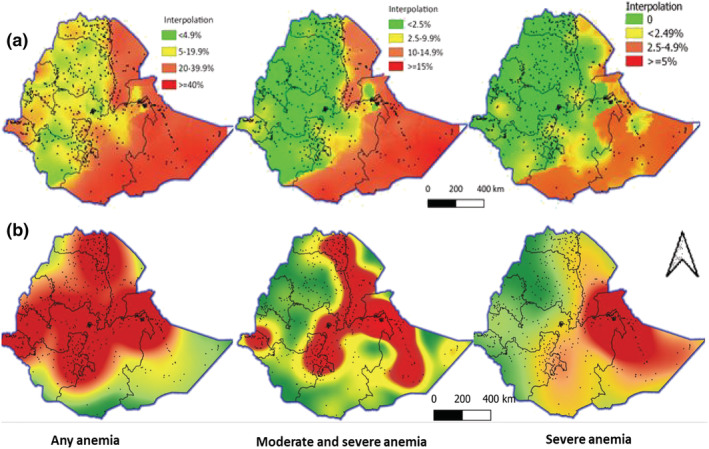
Predicted anaemia prevalence (a) and case‐load density (b) by severity, 2016

Figure 4a,b presents the predicted anaemia prevalence and case‐load density interpolated from measured areas. Contrasting findings, characterized by higher density of anaemia in low prevalence areas were obtained. Based on prevalence figures, anaemia was a serious public health concern in much of the eastern part of Ethiopia, but the density of anaemia cases was higher in low prevalence areas like the central and western part of Ethiopia. Moderate and severe cases were denser in much of the Great Rift Valley, Harar, part of Oromia, Afar and the Somali region. In contrast, both the density and prevalence figures showed that severe anaemia was a serious problem in large parts of the Somali and Harar regions, and in part of Afar and Oromia region.

Our adjusted multilevel model suggested that there is a strong association between anaemia and parity (birth in the last 5 years). Women who had >2 births in the last 5 years were 2.3 times more likely to have anaemia. Taking IFA tablets during pregnancy reduced the odds of being anaemic. Although being from a female‐headed household increased the odds of anaemia, residing in temperate zones (higher altitude) had lower risk of anaemia after adjusting haemoglobin values for altitude (Tables 1 and 2). Household wealth was inversely associated with anaemia, but WASH was not a significant predictor of anaemia. Regression analyses done separately for areas with anaemia prevalence above and below 20% showed similarities in factors associated with anaemia, except for taking IFA supplementation and low BMI found only associated in higher (>20%) and lower (<20%) prevalence areas, respectively (Table S3).

**Table 1:** Multilevel regression analyses identifying individual factors associated with anaemia, 2016 (*N* = 14,923)

Lower (individual) level characteristics	Sample (*n*)	Anaemia prevalence (%)	Any anaemia	
			COR	AOR
Birth interval (ref: ≥24 months)	6801	25.7	1	1
<24	1621	32	1.26 (1.11, 1.44)	0.96 (0.8, 1.15)
Currently pregnant (ref. no)	13,835	23.2	1	1
Yes	1088	29.1	1.24 (1.07, 1.44)	1.1 (0.9, 1.38)
Birth in last 5 years (ref. only 1)	12,070	21.2	1	1
Two	2429	32.6	1.58 (1.42, 1.76)	1.44 (1.23, 1.68)**
Above two	424	43.1	2.43 (1.95, 3.03)	2.32 (1.76, 3.05)**
Sex of household head (ref. male)	11,404	24.1	1	1
Female	3518	22.1	0.9 (0.82, 1)	1.26 (1.04, 1.53)**
Age (ref. 40–49)	2213	24.3	1	1
30–39	4078	25.5	1.06 (0.93, 1.2)	1.05 (0.85, 1.3)
20–29	5467	24.2	0.96 (0.8, 1.1)	0.97 (0.78, 1.21)
15–19	3165	19.9	0.75 (0.65, 0.9)	0.55 (0.23, 1.29)
Education (ref. secondary and above)	2464	15.8	1	1
Primary	5244	21.8	1.32 (1.14, 1.52)	0.74 (0.5, 1.08)
No education	7215	27.8	1.82 (1.58, 2.1)	0.93 (0.63, 1.37)
Currently breastfeeding (ref. no)	10,266	21.5	1	1
Yes	4657	28.3	1.33 (1.2, 1.4)	1.08 (0.93, 1.27)
BMI (ref. normal)	10,609	23.2	1	
Under weight	3154	26	1.1 (0.99, 1.2)	NI
Over weight	1133	21.3	0.99 (0.8, 1.2)	NI
Place of delivery (ref. health facility)	2326	22.5	1	1
Home	4890	30.4	1.27 (1.1, 1.47)	0.99 (0.82, 1.19)
IFA supplementation during pregnancy (ref. no)	4206	31.1	1	1
Yes	3108	23.6	0.79 (0.7, 0.9)	0.82 (0.7, 0.95)**
ANC (ref. >4)	2331	24.8	1	NS
<4	4983	29.4	1.1 (0.96, 1.3)	NS
Source of drinking water (ref. improved)	9635	21.6	1	1
Unimproved	4936	28.1	1.26 (1.13, 1.4)	1 (0.85, 1.18)
Toilet facility (ref. improved)	2241	20.4	1	1
Unimproved	7914	22	1.05 (0.9, 1.2)	0.92 (0.7, 1.2)
Open defecation	4414	28.7	1.3 (1.1, 1.5)	0.9 (0.67, 1.22)
Type of cooking fuel (ref. clean fuel)	888	15.9	1	1
Polluting fuel	13,670	24.3	1.46 (1.17, 1.8)	1.1 (0.66, 1.83)

Abbreviations: ANC, ante‐natal care; AOR, adjusted odds ratio; BMI, body mass index; COR, crude odds ratio; NI, not included because *P* > 0.2 in the unadjusted model; total sample size was 14,923.

**Table 2:** Multilevel regression analyses identifying community factors associated with anaemia, 2016 (*N* = 14,923)

Community level characteristics	Sample (*n*)	Anaemia prevalence (%)	Any anaemia	
			COR	AOR
Wealth quintile (ref. richest)	2519	17.4	1	1
Poorest	2519	34.3	2.1 (1.7, 2.5)	1.75 (1.24, 2.48)**
Second	2717	25.3	1.6 (1.4, 1.9)	1.38 (0.99, 1.91)
Middle	2891	23.7	1.6 (1.3, 1.9)	1.4 (1.03, 1.92)**
Fourth	2979	21.0	1.3 (1.1, 1.5)	1.17 (0.85, 1.6)
Residence (ref. urban)	3169	17.0	1	1
Rural	11,754	25.4	1.73 (1.4, 2.1)	1.5 (0.97, 2.32)
Ecology (ref. highland/>2300)	4177	23.2	1	1
Temperate (1501–2300 masl)	8691	21.5	0.91 (0.7, 1.1)	0.6 (0.46, 0.8)**
Lowland (501–1500 masl)	1886	31.6	1.9 (1.5, 2.5)	0.7 (0.47, 1.05)
Subtropical (<501 masl)	168	58.2	5.2 (3.3, 8.1)	1.66 (0.73, 3.74)
Distance to health facility (ref. not a big problem)	7367	21.7	1	NI
Big problem	7556	25.5	1.08 (0.98, 1.2)	NI
Region (ref. Addis Ababa)	825	16.0	1	1
Tigray	1073	19.7	1.35 (0.95, 1.92)	1.54 (0.68, 3.53)
Afar	119	44.7	4.73 (2.84, 7.86)	2.65 (0.92, 2.68)
Amhara	3645	17.2	1.07 (0.78, 1.48)	1.08 (0.49, 2.4)
Oromia	5422	27.3	2.02 (1.48, 2.77)	1.59 (0.72, 3.54)
Somalia	417	59.5	9 (6.14, 13.16)	5.39 (2.19, 13.2)**
Benishangul‐Gumuz	146	19.2	1.28 (0.75, 2.19)	1.28 (0.45, 3.62)
SNNPR	3124	22.5	1.44 (1.04, 1.98)	1.24 (0.23, 5.4)
Gambela	42	26.1	1.93 (0.87, 4.27)	1.13 (0.23, 5.34)
Harari	32	27.7	2.09 (0.87, 5)	3.25 (0.68, 15.4)
Dire Dawa	77	30.1	2.4 (1.3, 4.4)	3.85 (1.11, 13.39)**

Abbreviations: AOR, adjusted odds ratio; COR, crude odds ratio; NI, not included because *P* > 0.2 in the unadjusted model; the sample size for the analyses was 14,923.

## 4 DISCUSSION

In contrast to the WHA's ambition of reducing anaemia by 50% (relative to 2012) by 2025, the anaemia prevalence in WRA in Ethiopia has increased between 2011 and 2016. The prevalence of any, moderate and severe forms of anaemia was not equally distributed throughout the country, but instead showed clustering in a handful of hotspot areas. The rise in anaemia prevalence between 2011 and 2016 was for the most part related to the widening of existing hotspot areas, rather than the appearance of new hotspots. Noteworthy is the relatively low prevalence, but high absolute number of anaemia cases among WRA in densely populated areas.

The national prevalence of anaemia suggests that anaemia in WRA was a mild public health concern (<20%) in 2011, but increased to become a moderate public health concern in 2016 (WHO, 2011). However, such national level estimates mask within‐country differences, overestimating the spread and level of public health concern of anaemia in much of the west and central part of Ethiopia, while underestimating hotspot areas like Somali, Afar, Harar and part of Oromia. Our study has also showed that areas with low anaemia prevalence can still have higher anaemia density (caseloads), particularly in densely populated areas. Consequently, if decisions are not supported by subnational relative and absolute anaemia prevalence estimates, applying the WHO recommended cut‐off of 20% as a benchmark to prioritize interventions may not lead to efficient and effective use of available resources (WHO, 2014). Instead, our results highlight the value of subnational anaemia estimates and variation in programme design and policy decision‐making.

Our subnational mapping also triggered a number of questions related to which interventions are needed and how best should they be targeted for maximal impact? In light of limited resources, should interventions focus on increasing coverage to reach the maximum number of anaemia cases, or focus on reaching hotspots with high prevalence, yet with lower density of anaemia cases? This poses a coverage‐equity paradox, where increasing coverage may be more cost‐effective if prioritized to be implemented in more densely populated areas (e.g., west and central Ethiopia) than in more remote, less‐populated, areas (e.g., Harari and Somali). From an equity perspective, and considering the high prevalence of anaemia, more remote and less‐populated areas will still need to be prioritized. This coverage‐equity dilemma is however of a lesser concern if interventions are prioritized to focus only on the treatment of moderate and severe forms of anaemia, as both absolute and relative estimates converged, but also mild anaemia is associated with less serious health consequences than severe anaemia.

For prevention and treatment interventions to be effective and equitable, determining the aetiology of anaemia is critical. However, in many LMICs including Ethiopia, little is known about the aetiology of anaemia (Baye, 2019). Nutritional deficiencies including those of iron, folate, vitamin A and B12 may contribute, to a varying extent, to the anaemia prevalence in Ethiopia (EPHI, 2016). For example, both Afar and Somali regions have high prevalence of anaemia, but according to the Ethiopian National Micronutrient Survey (ENMS), iron deficiency (assessed by inflammation‐adjusted serum ferritin and serum transferrin concentrations) was relatively high in Somali (25%), but not in Afar (<10%) (EPHI, 2016). Besides, deficiencies of folate and vitamin B12 were found prevalent to a varying extent (EPHI, 2016). Such geographical differences in the contributors of anaemia remind us of the need for routine assessments of micronutrient status prior to implementing micronutrient interventions.

On the other hand, inflammation can lead to anaemia and may also coexist with iron deficiency in low income settings, where nutritional deficiencies and infections are common (Shaw & Friedman, 2011). From an evolutionary perspective, hypoferremia and increased production and activation of leukocytes serve as a host‐defense mechanism that is activated when facing infection or inflammatory events (Ganz, 2019). The resulting reduction in plasma iron concentration and transferrin saturation prevents the generation of non‐transferrin bound iron, a form of iron known to stimulate pathogenic microorganisms (Seyoum et al., 2021; Stefanova et al., 2017). Thus, indiscriminate iron supplementation to treat anaemia triggered by inflammation can override this host‐defense mechanism and lead to increases in the concentration of non‐transferrin bound iron, which has been linked to increased morbidity and mortality (Baye, 2019; Hurrell, 2010). Consequently, in settings where infections are widespread, addressing the primary cause of anaemia through infection treatment may be more effective than iron interventions (Ganz, 2019). This is of particular importance given the relatively high levels of inflammation in Dire Dawa and Somali, also identified as anaemia hotspots in this study (Challa et al., 2021). More importantly, given the protection conferred by mild and moderate iron deficiency, interventions may need to prioritize the treatment of moderate and severe forms over any anaemia (Wander et al., 2009).

Chronic exposure to inflammation/infections as seen in malaria‐endemic settings have been directly linked with anaemia, but have also been linked to more long‐term consequences like genetic polymorphisms (i.e., hemoglobinopathies) that can also cause anaemia (Goheen et al., 2017). Unfortunately, hemoglobinopathies have been rarely assessed, but are expected to be high in Africa (Modell & Darlison, 2008). The identified hotspot areas of anaemia are also malaria‐endemic areas, suggesting that the potential contribution of haemoglobin genetic disorders to the anaemia prevalence cannot be overruled, but evidence on this remains in‐existent. As iron‐based interventions remain the main anaemia prevention and treatment strategy, their effectiveness and possible adverse effects in the presence of hemoglobinopathies need to be evaluated (Coates et al., 2016; Wieringa et al., 2016).

On the other hand, underlying factors like birth interval, pregnancy and female‐headed households were associated with higher risk of anaemia. This is not surprising given the higher nutrient demands during pregnancy, which can be further exacerbated by multiple pregnancies, economic vulnerabilities, food insecurity and poverty (Chaparro & Suchdev, 2019; Wirth et al., 2017). Not surprisingly, taking IFA tablets during pregnancy reduced the risk of any anaemia (Oh et al., 2020), a finding that can also be partly a reflection of higher women (health) literacy and access to health care (Sendeku et al., 2020).

The present study has a number of limitations that need to be considered. First, although our study is based on nationally and regionally representative data from the last two rounds of DHS, the study remains cross‐sectional and thus does not allow causal inferences to be made. Although diet quality, nutrient intake and infections like malaria can be key determinants, they are not captured in the DHS and thus were not reflected in our multilevel regression models.

Notwithstanding the above limitations, this study provides a unique spatio‐temporal analysis of anaemia among WRA. To our knowledge, this is the first study, to provide subnational, local‐level, maps by anaemia prevalence by severity and density of caseloads. Such granular analyses can inform anaemia prevention and treatment interventions to be more precise, effective and locally adapted. Our study has identified a number of clusters of anaemia that require attention, but also highlighted that low prevalence areas can still have high absolute number of cases, particularly in populated areas, suggesting that both relative and absolute estimates are critical to determine where additional attention is needed. Our estimates of any, moderate and severe anaemia allow policymakers to prioritize interventions against resources available, but also calls for an urgent assessment of the aetiology of anaemia to increase effectiveness, safety and equity of interventions.

## CONFLICT OF INTEREST

None.

## CONTRIBUTIONS

KB, SC and AL conceived the study with inputs from BA and JB; BA and KB prepared and analysed the data; KB and BA wrote the paper with inputs from AL, SC and JB. All authors read and approved the final manuscript.

## Supporting information


**Figure S1:** Cold and hot‐spots of any, moderate and severe anemia
**Table S1** Clusters of any anemia among women of reproductive age, 2011 and 2016
**Table S2** Clusters of anemia among women of reproductive age by severity, 2016
**Table S3** Multi‐level regression analyses identifying individual factors associated with anemia in high (> 20%) and low (< 20%) prevalence areas, 2016

## Data Availability

The data that support the findings of this study are available on request from the https://dhsprogram.com/data/.
