# Supplementary material for: Subnational mapping for targeting anaemia prevention in women of reproductive age in Ethiopia: A coverage‐equity paradox
Source: Matern Child Nutr. 2021 Oct 8;20(Suppl 5):e13277. doi: 10.1111/mcn.13277 (PMC11258772; doi:10.1111/mcn.13277)
Supplement: Supplementary file 1 — Figure S1: Cold and hot‐spots of any, moderate and severe anemia Table S1 Clusters of any anemia among women of reproductive age, 2011 and 2016 Table S2 Clusters of anemia among women of reproductive age by severity, 2016 Table S3 Multi‐level regression analyses identifying individual factors associated with anemia in high (> 20%) and low (< 20%) prevalence areas, 2016 [file MCN-20-e13277-s001.docx]

**Supplement**


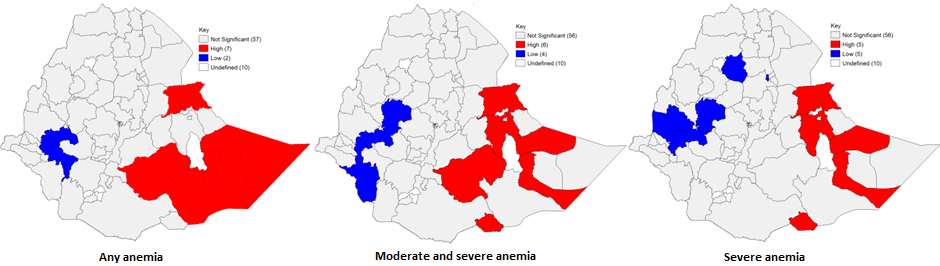


Figure S1: Cold and hot-spots of any, moderate and severe anemia

**Table S1** Clusters of any anemia among women of reproductive age, 2011 and 2016

| Cluster | **Radius (Km)** | | | **Relative risk (RR)** | | **Prevalence (%)** | | **P-value** | | |  |
| --- | --- | --- | --- | --- | --- | --- | --- | --- | --- | --- | --- |
|  | 2011 | | 2016 | 2011 | 2016 | 2011 | 2016 | | 2011 | 2016 | |
| 1 | 230 | 318 | | 1.8 | 2.4 | 33 | 61 | | <0.001 | <0.001 | |
| 2 | 322 | 265 | | 2.6 | 1.8 | 50 | 48 | | <0.001 | <0.001 | |
| 3 | 30 | 79 | | 2.9 | 1.7 | 57 | 47 | | <0.001 | <0.001 | |
| 4 | 60 | 82 | | 2.1 | 1.9 | 41 | 51 | | <0.002 | <0.002 | |
| 5 | 17 | 202 | | 2.5 | 1.7 | 50 | 47 | | <0.003 | <0.003 | |
| 6 | 70 | 26 | | 1.4 | 1.9 | 29 | 51 | | 0.3 | <0.001 | |
| 7 | 0 | 139 | | 2.9 | 1.4 | 58 | 37 | | >0.05 | >0.05 | |
| 8 | 0 | 0 | | 2.7 | 2.7 | 55 | 25 | | >0.05 | >0.05 | |
| 9 | 367 | 4 | | 1.6 | 1.7 | 31 | 46 | | >0.05 | >0.05 | |

Cluster numbers are those in the figure 2

| Cluster | **Radius** | | **Relative risk (RR)** | | **Prevalence (%)** | | **P-value** | |
| --- | --- | --- | --- | --- | --- | --- | --- | --- |
|  | Moderate & severe | Severe | Moderate & severe | Severe | Moderate & severe | Severe | Moderate & severe | Severe |
| 1 | 209 | 330 | 4.6 | 3.5 | 30 | 9 | <0.001 | <0.001 |
| 2 | 204 | 247 | 4.6 | 5 | 35 | 5 | <0.001 | <0.001 |
| 3 | 36 | 97 | 2.9 | 9.3 | 22 | 5 | <0.001 | <0.001 |
| 4 | 265 | 49 | 2.1 | 12 | 16 | 5 | <0.001 | 0.01 |
| 5 | 82 | 0 | 2.6 | 14.6 | 14 | 1.5 | <0.001 | 0.6 |
| 6 | 2 | 36 | 4.9 | 17 | 38 | 75 | <0.001 | 0.7 |
| 7 | 29 | 0 | 2.4 | 18 | 19 | 5 | 0.04 | 0.8 |
| 8 | 0 | 0 | 6.4 | 25 | 50 | 25 | 0.4 | 0.9 |

**Table S2** Clusters of anemia among women of reproductive age by severity, 2016

Cluster numbers are those in the figure 3

**Table S3** Multi-level regression analyses identifying individual factors associated with anemia in high (> 20%) and low (< 20%) prevalence areas, 2016

|  |  | **Areas with anemia prevalence** | | | | |
| --- | --- | --- | --- | --- | --- | --- |
|  |  | ≥ 20 % | | < 20 % | | |
|  | Sample (n) | COR | AOR | Sample (n) | COR | AOR |
| **Birth interval** (ref: >24 months) | 6801 | 1 | 1 | 5321 | 1 | 1 |
| <24 | 1621 | 1.26 (1.08,1.46) | 0.94 (0.77,1.15) | 1152 | 1.21(1.03,1.43) | 0.99(0.78,1.25) |
| **Currently pregnant** (Ref. No) | 13835 | 1 | 1 | 11027 | 1 | NI |
| Yes | 1088 | 1.17 (0.98,1.39) | 0.98 (0.75,1.28) | 793 | 1.08(0.9,1.3) | NI |
| **Birth in last 5 yrs** (Ref. only 1) | 12070 | 1 | 1 | 9914 | 1 | 1 |
| Two | 2429 | 1.57 (1.39,1.77) | 1.44 (1.20,1.72)** | 1654 | 1.55(1.35,1.77) | 1.43(1.18,1.73)** |
| Above two | 424 | 2.43 (1.90,3.11) | 2.32 (1.69,3.17)** | 252 | 2.56(1.92,3.42) | 2.35(1.66,3.33)** |
| **Sex of household head** (Ref. male) | 11404 | 1 | NI | 8876 | 1 | NI |
| Female | 3518 | 0.93 (0.82,1.04) | NI | 2944 | 0.94(0.83,1.05) | NI |
| **Age** (Ref. 40-49) | 2213 | 1 | 1 | 1815 | 1 | 1 |
| 30-39 | 4078 | 1.17 (1.01,1.37) | 1.11 (0.86,1.44) | 3178 | 1.02(0.88,1.19) | 1.11(0.86,1.43) |
| 20-29 | 5467 | 1.07 (0.92,1.25) | 0.99 (0.76,1.29) | 4319 | 0.87(0.75,0.99) | 0.94(0.72,1.23) |
| 15-19 | 3165 | 0.73 (0.61,0.86) | 0.59 (0.24,1.43) | 2508 | 0.78(0.66,0.91) | 1.18(0.46,3.03) |
| **Education** (Ref. Secondary and above) | 2464 | 1 | 1 | 2274 | 1 | 1 |
| Primary | 5244 | 1.20 (0.99,1.44) | 0.77 (0.47,1.27) | 4074 | 1.27(1.08,1.48) | 0.72(0.48,1.09) |
| No education | 7215 | 1.80 (1.50,2.15) | 1.03 (0.63,1.70) | 5472 | 1.72(1.48,2.02) | 0.99(0.66,1.49) |
| **Currently breastfeeding** (Ref. No) | 10266 | 1 | 1 | 8367 | 1 | 1 |
| Yes | 4657 | 1.36 (1.23,1.51) | 1.05 (0.88,1.27) | 3453 | 1.3(1.17,1.44) | 0.95(0.8,1.14) |
| **BMI** (Ref. Normal) | 10609 | 1 | NI | 8312 | 1 | 1 |
| underweight | 3154 | 1.02 (0.90,1.17) | NI | 2442 | 1.22(1.09,1.38) | 1.31(1.07,1.59)** |
| overweight | 1144 | 1.01 (0.81,1.24) | NI | 1050 | 1.01(0.85,1.22) | 0.85(0.6,1.2) |
| **Place of delivery** (Ref. Health facility) | 2326 | 1 | 1 | 2018 | 1 | 1 |
| Home | 4890 | 1.20(1.01,1.41) | 0.93 (0.75,1.15) | 3364 | 1.19(1.01,1.4) | 0.97(0.79,1.2) |
| **IFA supplementation during pregnancy** (Ref. No) | 3766 | 1 | 1 | 2864 | 1 | NI |
| Yes | 3547 | 0.76(0.66,0.88) | 0.77 (0.64,0.92)** | 2586 | 0.78(0.52,146) | NI |
| **ANC** (Ref. >4) | 5022 | 1 | 1 | 3733 | 1 | 1 |
| <4 | 2291 | 0.81(0.7,0.94) | 0.90 (0.76,1.07) | 1717 | 1.15(1.01,1.32) | 0.98(0.82,1.17) |
| **Source of drinking water** (Ref. improved) | 9635 | 1 | 1 | 7881 | 1 | 1 |
| Unimproved | 4936 | 1.30(1.16,1.47) | 1.02 (0.85,1.22) | 3627 | 1.15(1.01,1.32) | 0.93(0.76,1.13) |
| **Toilet facility** (Ref. improved) | 2241 | 1 | 1 | 2126 | 1 | 1 |
| Unimproved | 7914 | 0.97(0.81,1.16) | 0.99 (0.71,1.37) | 6053 | 0.98(0.83,1.15) | 0.84(0.62,1.14) |
| Open defecation | 4414 | 1.25(1.04,1.5) | 0.99 (0.70,1.4) | 3328 | 1.27(1.07,1.52) | 0.81(0.58,1.13) |
| **Type of cooking fuel** (Ref. clean fuel) | 888 | 1 | 1 | 856 | 1 | 1 |
| Polluting fuel | 13670 | 1.63(1.21,2.19) | 1.74 (0.91,3.34) | 10645 | 1.31(1.04,1.65) | 1.05(0.61,1.79) |
| **Wealth quintile** (Ref. Richest) | 3816 | 1 | 1 | 3556 | 1 | 1 |
| Poorest | 2519 | 2.05(1.7,2.47) | 1.64 (1.11,2.44)** | 1826 | 1.9(1.58,2.28) | 1.7(1.13,2.55)** |
| Second | 2717 | 1.56(1.3,1.89) | 1.36 (0.94,1.95) | 2010 | 1.39(1.15,1.67) | 1.22(0.83,1.79) |
| Middle | 2891 | 1.41(1.17,1.7) | 1.24 (0.87,1.78) | 2136 | 1.34(1.12,1.61) | 1.37(0.95,1.99) |
| Fourth | 2979 | 1.21(1.01,1.46) | 1.25 (0.87,1.78) | 2292 | 1.15(0.96,1.38) | 0.94(0.65,1.36) |
| **Residence** (Ref. Urban) | 3169 | 1 | 1 | 3052 | 1 | 1 |
| Rural | 11754 | 1.27(1.001,1.59) | 1.01 (0.57,1.82) | 8768 | 1.51(1.23,1.86) | 1.46(0.93,2.28) |
| **Ecology** (Ref. Highland/>2300) | 4177 | 1 | 1 | 3410 | 1 | 1 |
| Temperate (1501-2300 masl) | 8691 | 0.94(0.79,1.13) | 0.72 (0.54,0.94)** | 6606 | 0.92(0.76,1.12) | 0.74(0.55,0.99)** |
| Lowland (501-1500 masl) | 1886 | 1.73(1.38,2.17) | 0.86 (0.57,1.27) | 1636 | 2.16(1.7,2.76) | 1.55(1.07,2.25)** |
| Subtropical (<501 masl) | 168 | 3.01(2.1,4.5) | 1.86 (0.88,3.95) | 168 | 6.27(4.12,9.54) | 5.87(3.05,11.3)** |
| **Distance to health facility** (Ref. Not a big problem) | 7367 | 1 | NI | 5409 | 1 | 1 |
| Big problem | 7556 | 0.99 (0.89,1.11) | NI | 6411 | 0.86(0.77,0.96) | 1.01(0.83,1.22) |

AOR, adjusted odds ratio; COR, crude odds ratio; NI, not included because P>0.2 in the unadjusted model
